# Early elevation of high-sensitivity C-reactive protein as a predictor for cardiovascular disease incidence and all-cause mortality: a landmark analysis

**DOI:** 10.1038/s41598-023-41081-w

**Published:** 2023-08-29

**Authors:** Hye Sun Lee, Jun-Hyuk Lee

**Affiliations:** 1 Biostatistics Collaboration Unit, Department of Research Affairs, Yonsei University College of Medicine, Seoul, 03277 Republic of Korea; 2 Department of Family Medicine, Nowon Eulji Medical Center, Eulji University School of Medicine, Seoul, 01830 Republic of Korea; 3 Department of Medicine, Hanyang University Graduate School of Medicine, Seoul, 04763 Republic of Korea

**Keywords:** Cardiovascular diseases, Predictive markers, Prognostic markers, Chronic inflammation

## Abstract

We investigated the association between early elevation of high-sensitivity C-reactive protein (hsCRP) and cardiovascular disease (CVD) incidence, all-cause mortality, and CVD mortality. We analyzed 6567 participants from the Korean Genome and Epidemiology Study_Ansan_Ansung cohort between 2005 and 2018. The Kaplan–Meier curves and modified Cox regression by Fine and Gray were used to estimate hazard ratios (HRs) with 95% confidence intervals (CIs) for CVD incidence, all-cause mortality, CVD mortality, cancer mortality, and mortality from other causes. Landmark analyses were performed at the first (2007–2008) and second (2009–2010) follow-up periods, with early elevation defined as hsCRP > 2 mg/L. At the first and second landmark points, the early hsCRP elevation group had a higher incidence of CVD and all-cause mortality. At first landmark point, the adjusted HRs (95% CIs) were 1.37 (1.08–1.74) for incident CVD and 1.26 (1.04–1.53) for all-cause mortality, respectively. At second landmark point, the adjusted HRs in the early hsCRP elevation group were 1.45 (1.12–1.89) for incident CVD and 1.34 (1.10–1.63) for all-cause mortality, respectively. However, there were no significant differences in CVD mortality and cancer mortality between the groups. In conclusion, early elevation of serum hsCRP is a predictor of incident CVD and all-cause mortality. The timing of hsCRP increase is also a significant predictor of incident CVD, even considering the competing risk. Regular hsCRP testing may help monitor hsCRP trends and develop individualized treatment plans for CVD prevention.

## Introduction

Cardiovascular disease (CVD) is a major global health concern, which is responsible for the highest number of deaths worldwide. According to the results from global burden of disease study, CVD mortality steadily increased from 12.1 million in 1990 to 18.6 million in 2019 and accounted for 31.59% of all deaths in 2017^1–3^. Moreover, the number of incident cases of CVD increased by 77.12% between 1990 and 2019, rising from 31.31 million to 55.45 million^3^. Atherosclerosis, a major cause of CVD, is a chronic inflammatory condition that causes plaque buildup in artery walls, resulting in narrowed and hardened blood vessels^4,5^. Various inflammatory markers, such as high-sensitivity C-reactive protein (hsCRP), interleukin-6 (IL-6), and tumor necrosis factor-alpha (TNF-α), have been used to assess atherosclerosis risk and progression, in addition to traditional risk factors, such as cigarette smoking, hypertension (HTN), dyslipidemia, and diabetes mellitus (DM)^5–9^.

Serum hsCRP is one of the most widely used inflammatory biomarkers for atherosclerosis risk assessment in clinical practice^9,10^. Although there is firm evidence on the association of elevated serum hsCRP with the risk of CVD, most of previous studies have evaluated the risk of CVD development based on the cross-sectional hsCRP status^11–13^. However, cumulative exposure to elevated hsCRP also can predict the risk of CVD^14^. Wang et al.^14^ found that the risk of incident CVD was elevated in dose–response manner with the cumulative number of elevated hsCRP event. In this regard, there is possibility that serum hsCRP levels increase above a certain threshold over shorter time intervals also could be a predictor for the risk of CVD. There is evidence showing the impact of early elevation of inflammatory markers on adverse clinical outcomes^15,16^. Emsley et al.^15^ found that 6 patients with acute ischemic stroke exhibited early and sustained peripheral inflammatory responses, including elevations in CRP, erythrocyte sedimentation rate, and white blood cell count, particularly in the first 5–7 days after onset of symptoms, compared to 36 controls. These findings suggest that preexisting inflammatory conditions may contribute to stroke development. Additionally, Cuschieri et al.^16^ reported that early elevation in plasma IL-6 levels following severe injury was correlated with incident organ failure among 79 severely injured patients with shock. These findings suggest the importance of timing with regard to the elevation of inflammatory markers. From this perspective, we hypothesized that early elevation of hsCRP levels may also indicate a higher risk of developing CVD. Despite this assumption, there is currently limited evidence regarding the association between early hsCRP elevation and CVD risk.

Given that serum hsCRP levels can be monitored over time in clinical practice, clinicians may be able to reduce the risk of CVD by implementing early, intensive management of cardiovascular risk factors in individuals who exhibit early hsCRP elevation, given that it is associated with a higher risk of CVD incidence and mortality. Therefore, this study aimed to investigate whether early elevation of serum hsCRP level is associated with an increased risk of CVD incidence and mortality, using landmark analysis in a community-based, prospective cohort study.

## Results

### Baseline characteristics of the study population

Table 1 presents the baseline characteristics of the study population stratified by hsCRP status at different time points. In the full dataset, participants with at least one hsCRP > 2 mg/L event during the follow-up were more likely to be men, older, and have higher body mass index (BMI), waist circumference (WC), mean blood pressure (MBP), fasting plasma glucose (FPG), triglycerides, and prevalence of DM, HTN, and dyslipidemia and lower high-density lipoprotein (HDL) cholesterol than those with consistently hsCRP ≤ 2 mg/L. There were no significant differences in total energy intake, drinking status, or exercise status between the two groups. Similar trends were observed at both the first and second follow-up landmark points, except for serum low-density lipoprotein (LDL) cholesterol, which did not show a significant difference between the groups, with and without early hsCRP elevation.

**Table 1:** Baseline characteristics of the study population stratified by hsCRP status at different time points.

Variables	Full dataset			Landmark at 1st follow-up			Landmark at 2nd follow-up		
	Consistently hsCRP ≤ 2 mg/L (*n* = 3268)	hsCRP > 2 mg/L event at least once during the follow-up (*n* = 3299)	p*	hsCRP ≤ 2 mg/L until 1st f/u (*n* = 4225)	hsCRP > 2 mg/L event between baseline and 1st f/u (*n* = 1522)	*p*	hsCRP ≤ 2 mg/L until 2nd f/u (*n* = 3466)	hsCRP > 2 mg/L event between baseline and 2nd f/u (*n* = 1757)	*p*
	Mean ± SD or N (%)	Mean ± SD or N (%)		Mean ± SD or N (%)	Mean ± SD or N (%)		Mean ± SD or N (%)	Mean ± SD or N (%)	
Men	1450 (44.37)	1649 (49.98)	< 0.001	1953 (46.22)	764 (50.20)	0.008	1573 (45.38)	883 (50.26)	< 0.001
Age, years	54.512 ± 8.442	56.735 ± 8.690	< 0.001	54.874 ± 8.454	57.143 ± 8.642	< 0.001	54.668 ± 8.329	57.003 ± 8.704	< 0.001
BMI, kg/m^2^	24.154 ± 2.879	24.834 ± 3.164	< 0.001	24.294 ± 2.901	25.174 ± 3.315	< 0.001	24.261 ± 2.875	25.036 ± 3.255	< 0.001
Waist circumference, cm	82.871 ± 8.561	85.702 ± 8.785	< 0.001	83.403 ± 8.567	86.605 ± 8.877	< 0.001	83.199 ± 8.566	86.367 ± 8.793	< 0.001
Mean blood pressure, mmHg	91.370 ± 11.698	93.438 ± 11.637	< 0.001	91.796 ± 11.719	93.921 ± 11.484	< 0.001	91.682 ± 11.649	93.759 ± 11.677	< 0.001
Smoking status			< 0.001			< 0.001			< 0.001
Non-smoker	2158 (66.03)	1984 (60.18)		2746 (64.99)	897 (59.01)		2281 (65.81)	1051 (59.89)	
Ex-smoker	581 (17.78)	625 (18.96)		774 (18.32)	296 (19.47)		632 (18.23)	344 (19.60)	
Current smoker	529 (16.19)	688 (20.87)		705 (16.69)	327 (21.51)		553 (15.95)	360 (20.51)	
Current drinker	1545 (47.28)	1608 (48.74)	0.235	2042 (48.33)	733 (48.16)	0.909	1671 (48.21)	847 (48.21)	0.998
Regular exerciser	1594 (48.79)	1423 (43.13)	< 0.001	2055 (48.65)	655 (43.04)	0.0002	1714 (49.47)	754 (42.91)	< 0.001
FPG, mg/dL	90.674 ± 11.820	93.192 ± 15.802	< 0.001	90.828 ± 12.106	95.120 ± 18.506	< 0.001	90.862 ± 12.437	94.251 ± 17.602	< 0.001
Total cholesterol, mg/dL	191.829 ± 34.000	191.273 ± 34.650	0.512	191.104 ± 33.483	192.350 ± 35.191	0.231	191.063 ± 33.253	191.575 ± 35.420	0.614
Triglyceride, mg/dL	132.679 ± 89.952	148.159 ± 112.467	< 0.001	134.403 ± 91.380	155.215 ± 121.377	< 0.001	132.988 ± 92.387	152.032 ± 115.711	< 0.001
HDL cholesterol, mg/dL	45.093 ± 10.489	43.246 ± 9.818	< 0.001	44.778 ± 10.441	42.269 ± 9.201	< 0.001	44.854 ± 10.507	42.587 ± 9.415	< 0.001
LDL cholesterol, mg/dL	120.103 ± 31.083	118.396 ± 33.117	0.031	119.453 ± 31.383	119.038 ± 33.094	0.671	119.620 ± 31.235	118.582 ± 33.457	0.279
hsCRP, mg/L	0.584 ± 0.429	2.472 ± 4.728	< 0.001	0.639 ± 0.451	3.974 ± 6.249	< 0.001	0.616 ± 0.440	3.303 ± 5.866	< 0.001
Total energy intake, kcal/day	1803.527 ± 603.551	1778.548 ± 586.374	0.090	1799.798 ± 578.934	1778.187 ± 606.401	0.229	1804.049 ± 594.728	1770.719 ± 578.871	0.054
DM	439 (13.43)	590 (17.88)	< 0.001	561 (13.28)	345 (22.67)	< 0.001	445 (12.84)	368 (20.94)	< 0.001
HTN	1012 (30.97)	1281 (38.83)	< 0.001	1365 (32.31)	633 (41.59)	< 0.001	1087 (31.36)	712 (40.52)	< 0.001
Dyslipidemia	1509 (46.18)	1739 (52.71)	< 0.001	2000 (47.34)	855 (56.18)	< 0.001	1629 (47.00)	974 (55.44)	< 0.001

P-value were derived from Student’s t-test for the comparison of continuous variables or chi-square test for the comparison of categorical variables between two groups.

A P-value less than 0.05 was considered statistically significant.

*hsCRP* high-sensitivity C-reactive protein, *BMI* body mass index, *FPG* fasting plasma glucose, *HDL* high-density lipoprotein, *LDL* low-density lipoprotein, *DM* diabetes mellitus, *HTN* hypertension, *SD* standard deviation.

### Longitudinal association of elevation of hsCRP with incident CVD and all-cause mortality at different time points

During the median 11.84 years of the event accrual period, there were a total of 441 (6.72%) new-onset CVD cases. The CVD incidence rate per 1000 person-years was 6.05 in the group with hsCRP > 2 mg/L during the follow-up and 3.55 in the group with consistent hsCRP ≤ 2 mg/L. At the first landmark point, the CVD incidence rate per 1000 person-years was 5.50 in the group with early elevation of hsCRP and 3.36 in the group without early elevation. At the second landmark point, the CVD incidence rate per 1000 person-years was 4.42 in the group with early elevation of hsCRP and 2.60 in the group without early elevation. During the median 14.64 years of event accrual period, a total of 581 (8.85%) all-cause mortality cases and 114 CVD mortality cases were observed. The all-cause mortality rate per 1000 person-years was 6.90 in the group with hsCRP > 2 mg/L events at least once during the follow-up and 4.69 in the group with consistently hsCRP ≤ 2 mg/L. At the first landmark point, the all-cause mortality rate per 1000 person-years was 8.16 in the group with early elevation of hsCRP and 4.79 in the group without early elevation. At the second landmark point, the all-cause mortality rate per 1000 person-years was 7.87 in the group with early elevation of hsCRP and 4.30 in the group without early elevation.

**Figure 1:**
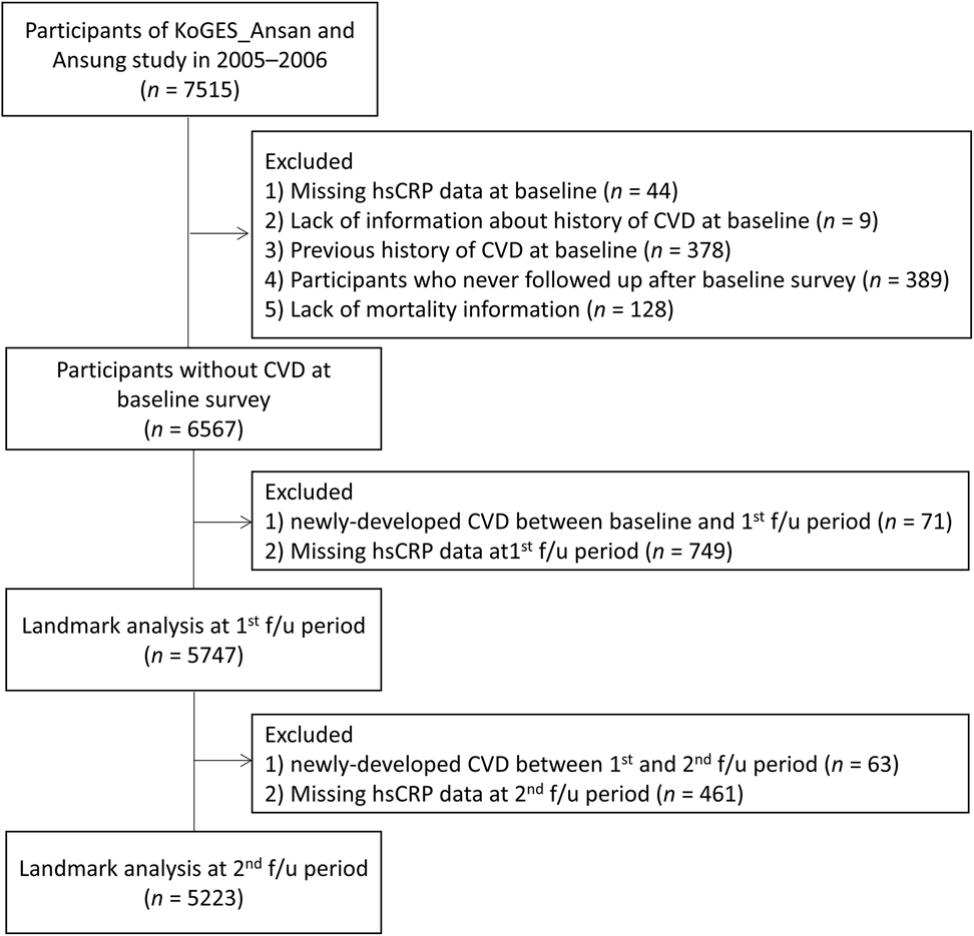
Kaplan–Meier curves showing the cumulative incidence rate of CVD and all-cause mortality according to the hsCRP elevation event at different time points, including full dataset (**A** incident CVD, **D** all-cause mortality), landmark at 1st follow-up (**B** incident CVD, **E** all-cause mortality), and landmark at 2nd follow-up: all-cause mortality (**C** incident CVD, **F** all-cause mortality). *hsCRP* high-sensitivity C-reactive protein, *CVD* cardiovascular disease.

Figure 1A–F show the Kaplan–Meier curves with log-rank tests for the cumulative incidence of CVD according to hsCRP elevation events at different time points. The group with hsCRP > 2 mg/L events at least once during follow-up had significantly higher cumulative rates of incident CVD than the group with consistently hsCRP < 2 mg/L (log-rank test *p* < 0.001 for all datasets; Fig. 1A–C). The same trends were observed for all-cause mortality (log-rank test *p* < 0.001 for all datasets; Fig. 1D–F).

Table 2 presents the results of modified Cox regression by Fine and Gray, examining the association between elevated serum hsCRP levels and incident CVD and all-cause mortality at different time points. For the full dataset, the group with hsCRP > 2 mg/L event at least once during follow-up period had a higher risk of incident CVD than the group with consistently hsCRP ≤ 2 mg/L [Hazard ratio (HR) 1.69, 95% confidence interval (CI) 1.39–2.05 in the univariable model; HR 1.45, 95% CI 1.19–1.77 in the multivariable model 3). The group with hsCRP > 2 mg/L event at least once during follow-up period had a higher risk of all-cause mortality in univariable model (HR 1.45, 95% CI 1.22–1.72), but the significance was not maintained in multivariable models. At the first follow-up landmark, the group with early elevation of hsCRP had a higher risk of incident CVD than the group without early elevation (HR 1.61, 95% CI 1.28–2.03 in the univariable model; HR 1.37, 95% CI 1.08–1.74 in the multivariable model 3). The significant higher risk for all-cause mortality in the group with early elevation of hsCRP, compared to the group without early elevation, was also shown. The corresponding HR (95% CI) was 1.69 (1.40–2.04) in univariable model and 1.26 (1.04–1.53) in multivariable model 3. Similarly, at the second follow-up landmark, the group with early elevation of hsCRP had a higher risk of incident CVD than the group without early elevation (HR 1.68, 95% CI 1.30–2.17 in the univariable model; HR 1.45, 95% CI 1.12–1.89 in the multivariable model 3). The significant higher risk for all-cause mortality in the group with early elevation of hsCRP, compared to the group without early elevation, was also shown. The corresponding HR (95% CI) was 1.83 (1.51–2.22) in univariable model and 1.34 (1.10–1.63) in multivariable model 3.

**Table 2:** Modified Cox regression by Fine and Gray for CVD incidence and all-cause mortality at different time points.

	Full dataset			Landmark at 1st f/u			Landmark at 2nd f/u		
	Consistently hsCRP ≤ 2 mg/L	hsCRP > 2 mg/L event at least once during the follow–up		hsCRP ≤ 2 mg/L until 1st f/u	hsCRP >2 mg/L event between baseline and 1st f/u		hsCRP ≤ 2 mg/L until 2nd f/u	hsCRP > 2 mg/L event between baseline and 2nd f/u	
Person–year	46,455	45,657		60,358	21,091		50,040	24,639	
Total cases	3268	3299		4225	1522		3466	1757	
Incident CVD cases	165	276		203	116		130	109	
Incident CVD rate per 1000 person–year	3.55	6.05		3.36	5.50		2.60	4.42	

	HR (95% CI)		*p*	HR (95% CI)		*p*	HR (95% CI)		*p*
Unadjusted	1 (reference)	1.69 (1.39–2.05)	< 0.001	1 (reference)	1.61 (1.28–2.03)	< 0.001	1 (reference)	1.68 (1.30–2.17)	< 0.001
Model 1	1 (reference)	1.48 (1.21–1.80)	< 0.001	1 (reference)	1.41 (1.11–1.79)	0.004	1 (reference)	1.50 (1.16–1.96)	0.002
Model 2	1 (reference)	1.47 (1.20–1.79)	< 0.001	1 (reference)	1.39 (1.10–1.77)	0.007	1 (reference)	1.49 (1.14–1.94)	0.003
Model 3	1 (reference)	1.45 (1.19–1.77)	< 0.001	1 (reference)	1.37 (1.08–1.74)	0.010	1 (reference)	1.45 (1.12–1.89)	0.006
Person–year	46,455	45,657		60,358	21,091		50,040	24,639	
Total cases	3268	3299		4225	1522		3466	1757	
All-cause mortality cases	218	315		289	172		215	194	
All-cause mortality rate per 1000 person–year	4.69	6.90		4.79	8.16		4.30	7.87	

	HR (95% CI)		*p*	HR (95% CI)		*p*	HR (95% CI)		*p*
Unadjusted	1 (reference)	1.45 (1.22–1.72)	< 0.001	1 (reference)	1.69 (1.40–2.04)	< 0.001	1 (reference)	1.83 (1.51–2.22)	< 0.001
Model 1	1 (reference)	1.11(0.93–1.32)	0.262	1 (reference)	1.35 (1.12–1.64)	0.002	1 (reference)	1.42 (1.16–1.72)	< 0.001
Model 2	1 (reference)	1.05(0.88–1.26)	0.558	1 (reference)	1.29 (1.07–1.57)	0.009	1 (reference)	1.37 (1.12–1.66)	0.002
Model 3	1 (reference)	1.06(0.89–1.26)	0.529	1 (reference)	1.26 (1.04–1.53)	0.018	1 (reference)	1.34 (1.10–1.63)	0.004

Model 1: Adjusted for age, sex, and BMI.

Model 2: Adjusted for variables used in Model 1 plus smoking status, current drinker, regular exerciser, and total energy intake.

Model 3: Adjusted for variables used in Model 2 plus DM, HTN, dyslipidemia.

*hsCRP* high-sensitivity C–reactive protein, *CVD* cardiovascular disease, *BMI* body mass index, *DM* diabetes mellitus, *HTN* hypertension, *HR* hazard ratio, *CI* confidence interval.

### Longitudinal association of elevation of hsCRP with CVD mortality, cancer mortality, and mortality from other causes at different time points

In the full dataset, the group with hsCRP levels exceeding 2 mg/L has a CVD mortality rate of 1.24, a cancer mortality rate of 2.76, and a rate of 3.33 for other causes, while the group consistently showing hsCRP levels at or below 2 mg/L demonstrates rates of 1.15 for CVD, 1.78 for cancer, and 1.91 for other causes. At the 1st landmark point, the group with early elevation of hsCRP exhibited a CVD mortality rate of 1.51, a cancer mortality rate of 3.06, and a rate of 3.79 for other causes. In contrast, the group without early elevation showed rates of 1.05 for CVD, 1.99 for cancer, and 1.96 for other causes. At the 2nd landmark point, the group with early elevation of hsCRP registered a CVD mortality rate of 1.42, a cancer mortality rate of 2.85, and a rate of 3.88 for other reasons, while those without the early elevation observed rates of 0.98 for CVD, 1.83 for cancer, and 1.58 for other causes.

**Figure 2:**
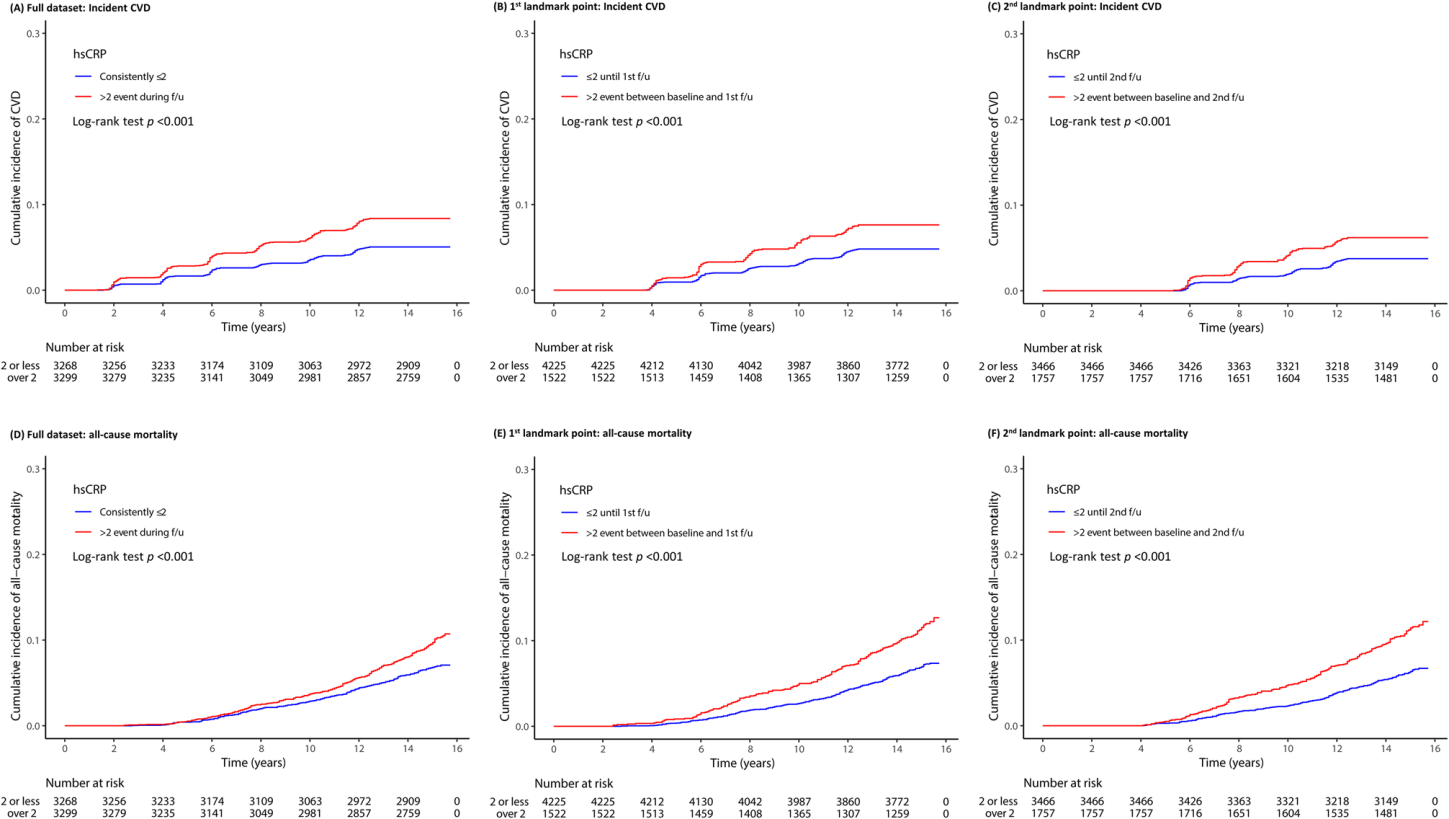
Kaplan–Meier curves showing the cumulative incidence rate of CVD mortality, cancer mortality, and mortality from other causes according to the hsCRP elevation event at different time points, including full dataset (**A** CVD mortality, **D** cancer mortality, **G** mortality from other causes), landmark at 1st follow-up (**B** CVD mortality, **E** cancer mortality, **H** mortality from other causes), and landmark at 2nd follow-up (**C** CVD mortality, **E** cancer mortality, **I** mortality from other causes). *hsCRP* high-sensitivity C-reactive protein, *CVD* cardiovascular disease.

Figures 2A–I present the Kaplan–Meier curves with log-rank tests for the cumulative rates of CVD mortality, cancer mortality, and mortality from other causes according to hsCRP elevation events at different time points. There were no significant differences in the cumulative incidence rates of CVD mortality between the two groups in the full dataset and at the landmark analyses at the first and second follow-ups (Fig. 2A–C). The group with hsCRP > 2 mg/L events at least once during follow-up had significantly higher cumulative rates of cancer mortality than the group with consistently hsCRP < 2 mg/L (log-rank test *p* = 0.002 for full dataset [Figs. 2D]; 0.005 for 1st landmark point [Fig. 2E]; 0.005 for 2nd landmark point [Fig. 2F]). The group with hsCRP > 2 mg/L events at least once during follow-up had significantly higher cumulative rates of mortality from other causes than the group with consistently hsCRP < 2 mg/L (log-rank test *p* < 0.001 for all datasets; Fig. 2G–I).

Table 3 shows the results of the modified Cox regression by Fine and Gray, examining the association between elevated serum hsCRP levels and CVD mortality, cancer mortality, and mortality from other causes at different time points. For the full dataset, the adjusted HR (95% CI) in the group with hsCRP > 2 mg/L events at least once during the follow-up, compared to the group with consistent hsCRP ≤ 2 mg/L, was 0.72 (0.49–1.06) for CVD mortality, 1.19 (0.90–1.58) for cancer mortality, and 1.28 (0.99–1.66) for mortality from other causes, respectively, in multivariable model 3. At the first landmark point, the adjusted HR (95% CI) in the group with early elevation of hsCRP, compared to the group without early elevation, was 0.96 (0.63–1.47) for CVD mortality, 1.18 (0.87–1.61) for cancer mortality, and 1.44 (1.08–1.92) for mortality from other causes, respectively, in multivariable model 3. At the second landmark point, the adjusted HR (95% CI) in the group with early elevation of hsCRP, compared to the group without early elevation, was 0.92 (0.60–1.42) for CVD mortality, 1.17 (0.85–1.61) for cancer mortality, and 1.80 (1.34–2.43) for mortality from other causes, respectively, in multivariable model 3.

**Table 3:** Modified Cox regression by Fine and Gray for CVD mortality, cancer mortality, and mortality from other causes at different time points.

	Full dataset			Landmark at 1st f/u			Landmark at 2nd f/u		
	Consistently hsCRP ≤ 2 mg/L	hsCRP > 2 mg/L event at least once during the follow–up		hsCRP ≤ 2 mg/L until 1st f/u	hsCRP > 2 mg/L event between baseline and 1st f/u		hsCRP ≤ 2 mg/L until 2nd f/u	hsCRP > 2 mg/L event between baseline and 2nd f/u	
Person–year	47,677	47,741		61,688	21,873		50,769	25,280	
Total cases	3268	3299		4225	1522		3466	1757	
CVD mortality cases	55	59		65	33		50	36	
CVD mortality rate per 1000 person–year	1.15	1.24		1.05	1.51		0.98	1.42	

	HR (95% CI)		*p*	HR (95% CI)		*p*	HR (95% CI)		*p*
Unadjusted	1 (reference)	1.06(0.74–1.53)	0.748	1 (reference)	1.41(0.93–2.15)	0.106	1 (reference)	1.43(0.93–2.19)	0.105
Model 1	1 (reference)	0.77(0.53–1.13)	0.182	1 (reference)	1.05(0.68–1.61)	0.832	1 (reference)	1.02(0.66–1.58)	0.927
Model 2	1 (reference)	0.73(0.50–1.07)	0.103	1 (reference)	0.99(0.64–1.52)	0.953	1 (reference)	0.95(0.62–1.47)	0.825
Model 3	1 (reference)	0.72(0.49–1.06)	0.099	1 (reference)	0.96(0.63–1.47)	0.848	1 (reference)	0.92(0.60–1.42)	0.716
Person–year	47,677	47,741		61,688	21,873		50,769	25,280	
Total cases	3268	3299		4225	1522		3466	1757	
Cancer mortality cases	85	132		123	67		93	72	
Cancer mortality rate per 1000 person–year	1.78	2.76		1.99	3.06		1.83	2.85	

	HR (95% CI)		*p*	HR (95% CI)		*p*	HR (95% CI)		*p*
Unadjusted	1 (reference)	1.55 (1.18–2.04)	0.002	1 (reference)	1.53 (1.13–2.06)	0.005	1 (reference)	1.54 (1.13–2.10)	0.006
Model 1	1 (reference)	1.25(0.95–1.65)	0.113	1 (reference)	1.26(0.93–1.70)	0.141	1 (reference)	1.24(0.90–1.69)	0.185
Model 2	1 (reference)	1.19(0.90–1.58)	0.215	1 (reference)	1.21(0.89–1.65)	0.219	1 (reference)	1.21(0.88–1.66)	0.244
Model 3	1 (reference)	1.19(0.90–1.58)	0.214	1 (reference)	1.18(0.87–1.61)	0.295	1 (reference)	1.17(0.85–1.61)	0.321
Person–year	47,677	47,741		61,688	21,873		50,769	25,280	
Total cases	3268	3299		4225	1522		3466	1757	
Other mortality cases	91	159		121	83		80	98	
Other mortality rate per 1000 person–year	1.91	3.33		1.96	3.79		1.58	3.88	

	HR (95% CI)		*p*	HR (95% CI)		*P*	HR (95% CI)		*p*
Unadjusted	1 (reference)	1.74 (1.35–2.26)	< 0.001	1 (reference)	1.92 (1.45–2.54)	< 0.001	1 (reference)	2.46 (1.83–3.31)	< 0.001
Model 1	1 (reference)	1.31 (1.01–1.70)	0.041	1 (reference)	1.51 (1.14–2.01)	0.004	1 (reference)	1.88 (1.39–2.53)	< 0.001
Model 2	1 (reference)	1.27(0.98–1.65)	0.071	1 (reference)	1.47 (1.11–1.96)	0.008	1 (reference)	1.84 (1.37–2.48)	< 0.001
Model 3	1 (reference)	1.28(0.99–1.66)	0.063	1 (reference)	1.44 (1.08–1.92)	0.013	1 (reference)	1.80 (1.34–2.43)	< 0.001

Model 1: Adjusted for age, sex, and BMI.

Model 2: Adjusted for variables used in Model 1 plus smoking status, current drinker, regular exerciser, and total energy intake.

Model 3: Adjusted for variables used in Model 2 plus DM, HTN, dyslipidemia.

*hsCRP* high-sensitivity C-reactive protein, *CVD* cardiovascular disease, *BMI* body mass index, *DM* diabetes mellitus, *HTN* hypertension, *HR* hazard ratio, *CI* confidence interval.

## Discussion

In this study, early elevation of serum hsCRP levels was associated with an increased risk of incident CVD, independent of traditional risk factors, such as cigarette smoking, DM, HTN, and dyslipidemia. The clinical significance of these findings is noteworthy, as the association remained significant even after considering all-cause mortality as a competing risk. This emphasizes the importance of monitoring longitudinal changes in hsCRP levels for CVD risk assessment. This finding is consistent with previous evidence demonstrating the impact of the early elevation of inflammatory markers on adverse clinical outcomes^12,13^. Our results also corroborate the well-established association between elevated hsCRP levels and increased risk of CVD^8–11^. However, our study extends the current knowledge by demonstrating the importance of longitudinal changes in hsCRP levels in predicting CVD risk.

The JUPITER trials focused on individuals with elevated levels of serum hsCRP (≥ 2 mg/L) but LDL cholesterol levels below 130 mg/dL^17^. The JUPITER trials demonstrated that early identification and management of individuals with elevated serum hsCRP levels, through statin therapy, can significantly reduce the incidence of CVD. This underscores the importance of routine screening for hsCRP levels in individuals at risk of CVD and the potential benefits of early intervention in this population. In this study, serum LDL cholesterol levels were < 130 mg/dL in individuals with early elevation of serum hsCRP at the first and second landmark points. Our findings support previous ones in the field and emphasize the importance of early identification and management of individuals with elevated serum hsCRP levels.

Although our study found a significant association between early elevated hsCRP levels and the risk of incident CVD and all-cause mortality, no significant association was observed with CVD-specific mortality while significant association was observed with mortality from other causes. One possible explanation for this is the presence of competing risks which were not considered in this study. Individuals with elevated hsCRP levels may have a higher risk of dying from other causes, such as infectious diseases or DM, which may obscure the association with CVD mortality. Lee et al.^18^ suggested that the association of serum hsCRP with cancer and CVD mortality risk can be attenuated by sex and comorbidities. They analyzed data from a total of 41,070 men and 81,011 women aged 40 years or older, with a follow-up period of 6.8 years, and found that serum hsCRP and risk of all-cause mortality exhibited a strong linear correlation, and that the association between hsCRP and cancer mortality risk was not observed in women with comorbidities, while the relationship with CVD mortality risk was mainly seen in men with comorbidities^18^. In addition, individuals at high risk for CVD are more likely to receive aggressive medical treatment, such as lipid-lowering therapies, antidiabetic medications, antihypertensive medications, or antithrombotic medications, which can reduce their risks of CVD-related death. The insufficient sample size and follow-up duration in our study might also have contributed to the insignificant association between early elevated hsCRP levels and CVD mortality. Further research with a larger sample size and longer follow-up period may potentially reveal a more significant association.There are several possible explanations for our results. First, it is important to consider that lifestyle factors, such as poor diet, sedentary behavior, and stress, can contribute to the early elevation of hsCRP levels. Prolonged exposure to these factors may exacerbate the inflammatory response and lead to a higher risk of developing CVD. This elevated risk can manifest as increased hsCRP levels, which act as markers of chronic inflammation^19^. In particular, considering that the only significant proportional difference between the two groups was in terms of current smokers, cigarette smoking might have acted as a major contributor to the elevation of serum hsCRP in this study. Second, genetic factors may also play some roles in the development of early elevation of serum hsCRP level^20–23^. Fransén et al.^20^ found strong associations between CRP and rs3091244, rs1800947, rs1130864, and rs1205 genotypes in women and rs1800947 in men. In addition, Gholami et al.^21^ also also found a significant relationship between hsCRP and genetic risk score, including single nucleotide polymorphisms in rs17782313, rs3807992, and rs2287161. In this study, certain polymorphisms might have contributed to chronic low-grade inflammation in individuals with early elevation of serum hsCRP levels, resulting in faster attainment of hsCRP levels > 2 mg/L. However, we were unable to confirm the existence of a specific genetic polymorphism because we did not conduct a genome-wide association study (GWAS). To obtain a more comprehensive and robust analysis, further GWAS is necessary.

This study had several key limitations. First, landmark analysis assumed that the onset of hsCRP levels > 2 mg/L would persist in the future. However, this approach might not completely exclude cases with transient hsCRP elevation, which might not have a sustained impact on CVD risk. Second, we did not account for potential changes in medication use during the follow-up period, such as lipid-lowering agents, antihypertensive medications, or antidiabetic medications, which may affect the outcomes of interest. Third, we did not consider the potential influence of other inflammatory markers on the outcomes of interest. Furthermore, we did not analyze temporal trends of changes in serum hsCRP levels or their associations with the development of incident CVD or mortality. Future studies verifying the association between serum hsCRP trajectories and incident CVD and mortality should be performed. Finally, although we utilized expert examiners to verify each case through comprehensive, face-to-face interviews, it's worth noting the potential for information bias in characterizing incident CVD events due to the reliance on self-reported questionnaires. Nevertheless, our methodology's credibility is supported by a previous study that reported a 93% match between self-reported diagnoses and those confirmed by physician reviews of medical records^24^. Nevertheless, we firstly verified early elevation of hsCRP as a risk factor for incident CVD and all-cause mortality. Additionally, to the best of our knowledge, this is the first study to conduct a competing risk analysis of mortality in relation to hsCRP and incident CVD risk.

In conclusion, early elevation of serum hsCRP levels is associated with a higher risk of developing CVD and all-cause mortality. Our findings suggest that the time to hsCRP increase is also important in predicting the occurrence of CVD, even considering competing risk. Therefore, in clinical practice, regular hsCRP testing can help monitor the trend of hsCRP increase and establish personalized treatment plans for preventing CVD in patients. This strategy may help healthcare providers identify individuals at an increased risk of CVD and take necessary steps to prevent the development of CVD and mortality.

## Methods

### Study population

We conducted an analysis using data from the Korean Genome and Epidemiology Study (KoGES)_Ansan_Ansung cohort, a community-based prospective cohort study conducted by the Korea Disease Control and Prevention Agency^25^. The cohort was established in 2001–2002, and participants were surveyed biennially until the eighth follow-up in 2017–2018. The study population consisted of community-dwelling adults aged 40–69 years who had lived in the Ansan (urban) and Ansung (rural) areas for at least 6 months.

**Figure 3:**
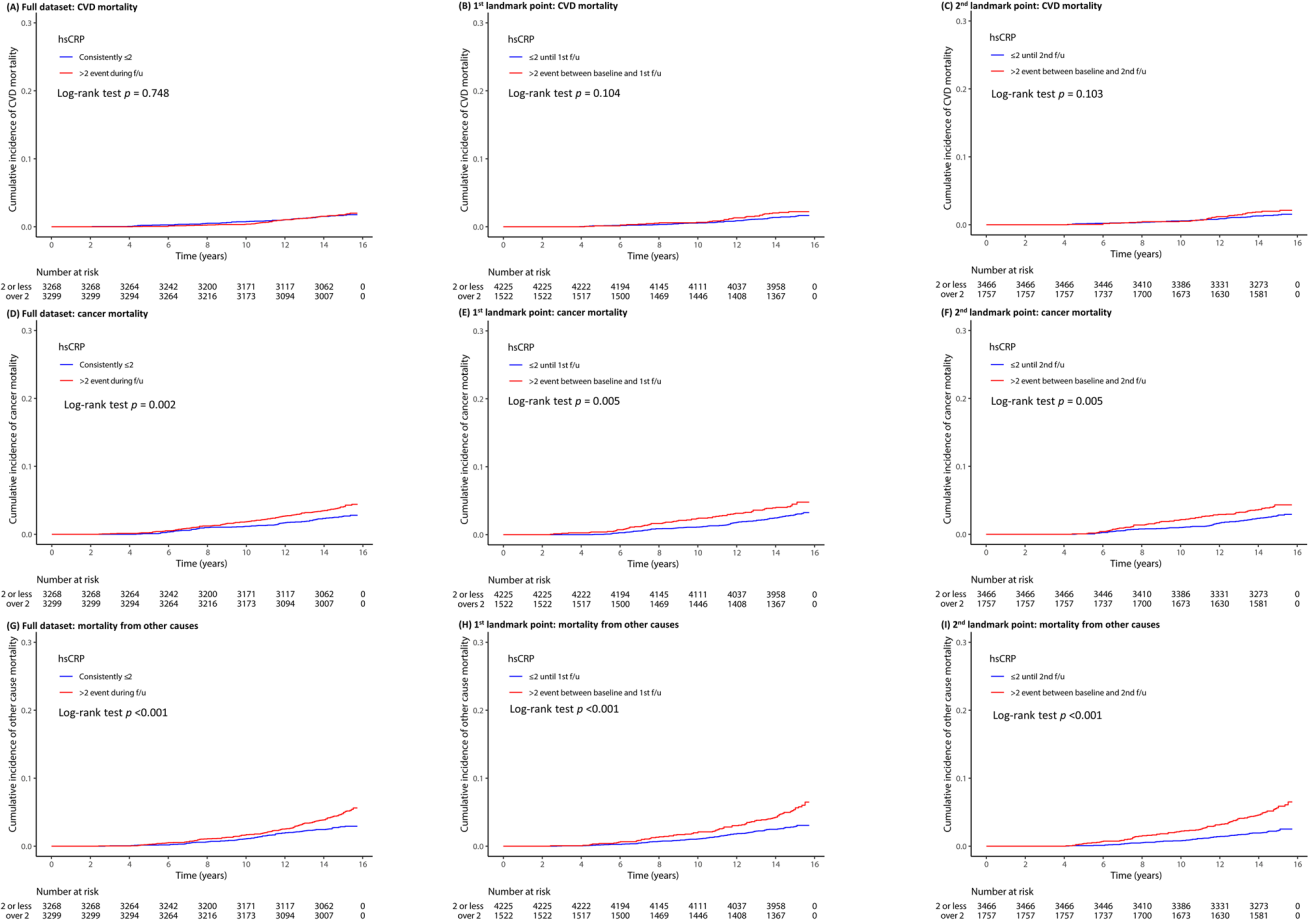
Flowchart showing the selection of the study population. *KoGES* Korea Genome and Epidemiology Study, *hsCRP* high-sensitivity C-reactive protein, *CVD* cardiovascular disease.

The analysis was limited to data collected between 2005–2006 and 2017–2018 due to the fact that hsCRP was not measured in 2001–2002, and there was a high proportion of missing hsCRP values in 2003–2004 (66%, 5,660 out of 8,603). The baseline data for the analysis were set as the data collected in 2005–2006, as illustrated in Fig. 3. Among the 7515 participants at baselines, we excluded those with missing serum hsCRP data (*n* = 44), those lacking information regarding CVD history (*n* = 9), those with CVD history at baseline (*n* = 378), those who did not follow up during the follow-up period (*n* = 389), and those with missing mortality information (*n* = 128). In the end, 6567 participants were included in the analysis.

Immortal time bias could lead to underestimation of the rate of clinical outcomes in patients who achieve the surrogate endpoints after the baseline and overestimation in those who fail to achieve these endpoints, which means that the real clinical outcomes may not be accurately reflected due to the skewness caused by immortal time bias^26,27^. To investigate the effect of early elevation of hsCRP as a risk factor for the development of CVD and account for the potential effect of immortal time bias, we performed landmark analyses^28^. Furthermore, to ensure sufficient time to accumulate cases of CVD incidence after the landmark points, we established the 1st and 2nd follow-ups as the landmark time points. at the first (2007–2008) and second (2009–2010) follow-up periods subsequent to the establishment of the baseline (2005–2006). The number of participants at the first landmark point was 5747, and the number at the second landmark point was 5223.

The KoGES_Ansan_Ansung cohort protocol was reviewed and approved by the Institutional Review Board (IRB) of the Korea Disease Control and Prevention Agency. All the participants read and signed a written informed consent form. The study protocol conformed to the ethical guidelines of the 1964 Declaration of Helsinki and its later amendments. This study was approved by the IRB of the Nowon Eulji Medical Center (IRB number: 2022-12-010).

### Data collection

Personal medical history, dietary habits, smoking status, alcohol consumption, physical activity, monthly household income, and educational level were obtained from each participant using questionnaires. Personal medical history included HTN, DM, dyslipidemia, myocardial infarction, angina pectoris, peripheral artery disease, and ischemic stroke. The age of the patient at the time of diagnosis of each disease was also obtained. The total energy intake (kcal/day) was calculated using a 103-item food frequency questionnaire. Participants were categorized into: never smokers, former smokers, or current smokers; current drinkers or non-drinkers; and regular exercisers or non-exercisers. After at least 8 h of fasting, blood samples were collected from each participant. FPG, serum insulin, total cholesterol, triglyceride, and HDL cholesterol levels were analyzed. In cases of serum triglyceride levels < 400 mg/dL, serum LDL cholesterol was calculated using the Friedewald formula.

### Definition of serum hsCRP elevation

Serum hsCRP levels were measured using an immunoradiometric assay (ADVIA 165; Siemens, Erlangen, Germany). We defined a serum hsCRP elevation event as the first occurrence during the follow-up period when hsCRP levels exceeded 2 mg/L, based on the highest concordance index in the preliminary analysis and optimal cut-off points, considering previous patient history as well^12,29,30^. At baseline, participants were classified into one group that had hsCRP levels > 2 mg/L at least once during follow-up and another group that had consistent hsCRP levels ≤ 2 mg/L. If hsCRP > 2 mg/L was detected at each landmark point, it was defined as an early elevation of hsCRP. The participants were classified into the early elevation hsCRP and non-early elevation hsCRP groups.

### Clinical outcomes

The primary outcome of interest was incident CVD, which we defined as newly developed myocardial infarction, angina pectoris, peripheral artery disease, and/or ischemic stroke. When a participant reported an incident CVD event on the personal medical history questionnaire, well-trained examiners conducted in-depth personal interviews to confirm the case. The follow-up time was defined as the time interval from the first follow-up period to the occurrence of a new-onset CVD event. The secondary outcomes were all-cause and CVD mortalities. To ascertain these outcomes, we linked the personal identification key code generated by the KoGES with national data sources, including death records from the Korea National Statistical Office. Information regarding cause-of-death and date-of-death of the participants was tracked from January, 2001 to December, 2020. The underlying causes of death were determined based on the Korean Standard Classification of Diseases codes listed in the National Death Index, and CVD mortality was defined according to the International Classification of Diseases 10th revision (ICD-10) codes I00–I99, while cancer mortality was determined using the ICD-10 codes C00–C97. Mortality from other causes refers to deaths caused by conditions other than CVD and cancer.

### Statistical analysis

We analyzed data separately using the full dataset, landmarks at the first follow-up, and landmarks at the second follow-up. Data are presented as mean ± standard deviation for continuous variables and number (%) for categorical variables. Student’s t-tests were used to compare differences in continuous variables, and chi-squared test was used to compare differences in categorical variables between the two groups at baseline and the first and second follow-up periods.

We compared the risk of CVD incidence and mortality between the group with serum hsCRP > 2 mg/L at least once during follow-up and those without. The Kaplan–Meier curves with log-rank tests were used to present the cumulative rates of incident CVD, all-cause mortality, CVD mortality, cancer mortality, and mortality from other causes between the two groups. Given the increasing recognition of the influence of competing risks in the development of prognostic models^31^, we used modified Cox regression by Fine and Gray to estimate hazard ratios (HRs) with 95% confidence intervals (Cis) for incident CVD, and death was considered as a competing risk. We did not count a mortality case if there was a CVD incidence event before death (48 cases). Furthermore, we also conducted competing risk analysis on all-cause mortality, stratifying it into CVD mortality, cancer mortality, and other-cause mortality. In the multivariable model, we included age, sex, BMI, smoking status, drinking status, physical activity, total energy intake, MBP, FPG, and serum LDL cholesterol level.

All statistical analyses were conducted using R software (version 4.1.1; R Foundation for Statistical Computing, Vienna, Austria). Statistical significance was set at p < 0.05.

### Ethcal approval

The KoGES_Ansan_Ansung cohort protocol was reviewed and approved by the Institutional Review Board (IRB) of the Korea Disease Control and Prevention Agency. All the participants read and signed a written informed consent form. The study protocol conformed to the ethical guidelines of the 1964 Declaration of Helsinki and its later amendments. This study was approved by the IRB of the Nowon Eulji Medical Center (IRB number: 2022-12-010).

## Data Availability

The dataset used in this study are available from the Korea Disease Control and Prevention Agency with permission and can be accessed at https://www.kdca.go.kr/contents.es?mid=a40504020100.

## Abbreviations

CVD: Cardiovascular disease
hsCRP: High-sensitivity C-reactive protein
IL-6: Interleukin-6
TNF-α: Tumor necrosis factor-alpha
HTN: Hypertension
DM: Diabetes mellitus
BMI: Body mass index
WC: Waist circumference
MBP: Mean blood pressure
FPG: Fasting plasma glucose
HDL: High-density lipoprotein
LDL: Low-density lipoprotein
HR: Hazard ratio
CI: Confidence interval
GWAS: Genome-wide association study
KoGES: Korean Genome and Epidemiology Study
IRB: Institutional Review Board
